# Service provision of non-invasive prenatal testing for Down syndrome in public and private healthcare sectors: a qualitative study with obstetric providers

**DOI:** 10.1186/s12913-018-3540-9

**Published:** 2018-09-21

**Authors:** Olivia Miu Yung Ngan, Huso Yi, Shenaz Ahmed

**Affiliations:** 1 CUHK Centre for Bioethics, Faculty of Medicine, The Chinese University of Hong Kong, Shatin, New Territories Hong Kong SAR; 2 Saw Swee Hock School of Public Health, National University of Singapore and National University Health System, Tahir Foundation Building, 12 Science Drive 2, #09-01W, Singapore, 117549 Singapore; 3 Leeds Institute of Health Sciences, School of Medicine, University of Leeds, Leeds, LS2 9NL UK

**Keywords:** Non-invasive prenatal testing (NIPT), Down syndrome screening, Service provision, Healthcare delivery, Implementation, Qualitative study, Hong Kong

## Abstract

**Background:**

Cell-free fetal DNA sequencing based non-invasive prenatal testing (NIPT) for Down syndrome (DS) has become widely available. In Hong Kong, obstetric providers in the public sector refer women identified at high risk of having a child with Down syndrome to obstetric providers in the private sector for NIPT. Little is known about how the NIPT has been adopted in the public sector where DS screening is provided for free of charge. The study aimed to identify the factors influencing providers’ role enactment, such as consultation and referral, in the service provision of NIPT for DS in public and private healthcare sectors.

**Methods:**

In-depth interviews were conducted with 20 obstetric providers offering NIPT in Hong Kong. Thematic narrative analysis was used to identify (i) the factors considered by participants when referring women for NIPT for Down syndrome in public and private healthcare sectors and (ii) their perceptions of the need to integrate NIPT into the current public antenatal service.

**Results:**

Participants raised concerns about the lack of transparent referral guideline between public and private sectors for NIPT. Public obstetric providers reported little obligation to provide women with much information about risks and benefits of NIPT as it was not provided by public sectors. Some private providers assumed that women referred from the public sector had already received sufficient information about NIPT. The providers were also concerned about potential application of NIPT for further detection without regulation.

**Conclusions:**

Although the providers had good knowledge of clinical advantages of NIPT over conventional screening, they were uncertain about how to introduce NIPT to women. Guidelines are necessary to enable better coordination of public and private sectors services to enable women to make informed choices about the uptake of NIPT.

## Background

Non-invasive prenatal testing (NIPT) based on sequencing cell-free fetal DNA in maternal plasma [1] has marked increase in the accuracy of screening for Down Syndrome (DS) in antenatal care settings. Research evidence substantially reported that NIPT showed consistent validity among the general and high-risk population, with an overall higher sensitivity of 99.7% and lower of 0.04% [2] than the routine first trimester combined screening (90% for a 5% false-positive rate) [3], which is offered for free as a routine check-up in many countries [4]. Based on the latest evidence of analytical and clinical validity, professionals’ societies in obstetrics have recommended NIPT as a primary screening for all pregnant women regardless of age and risk status [5]. To exclude false-positives, a positive NIPT result should always be confirmed with invasive prenatal diagnoses (IPD), such as amniocentesis or chorionic villus sampling that carry a procedure-related risk of up to 1% [6]. Due to the absence of the procedural risk NIPT has become widely available [7].

In prenatal testing, informed decision-making is essential to patient-centred care [8]. However, women may not be sufficiently informed about DS screening during the consultation, partly because obstetric care providers prioritise the safety and validity of testing over women’s needs and understanding of it [9]. The availability of NIPT raises concerns about diminishing informed consent. Obstetric providers report less need for consent procedures for NIPT compared to IPD [10, 11], although some women perceived NIPT as diagnostic testing [12]. Studies report women’s preference of deferring decision-making to obstetricians with the assumption that experts choose the best option for them [13]. The roles of obstetric providers in the public sector are more important in the consultation of NIPT because the test is new, and women have to pay out of pocket. Women, however, report little opportunity to discuss NIPT with obstetric providers and report layman resources (e.g., press media, pamphlets by testing companies) as more useful [7]. However, these resources provide selective information based on commercial interests and did not always meet clinical and ethical standards [14]. The literature of clinical use of NIPT highlighted the critical role by obstetric providers to ensure women receive accurate information before test uptake.

Since the launch of NIPT in late 2011, studies have explored attitudes towards NIPT among obstetric providers and patients [13, 15–21]. Overall, the providers reported preference for NIPT over conventional DS screening due perceived clinical advantages of lower numbers of false-positive results and reduce maternal anxiety [11]. Implementation of NIPT in the public health system of Hong Kong, providing free DS screening and diagnosis is challenging. NIPT has been rapidly adopted in practice; for example, a private clinic reported about 80% antenatal women chose NIPT [22]. A local study of 651 pregnant women reported about two-thirds failed to distinguish NIPT from diagnostic tests requiring confirmation of positive cell-free fetal DNA results [23]. It is unclear when and how obstetricians working in the public sector need to refer women for the privately available NIPT. Studies reported that public sectors are constrained by a higher degree of governing formalisation that limits the extent of participation in the decision-making process with patients [24], while private sectors place emphasis on economic rewards that healthcare services are observed as business discourse [25]. Different organisational structures and values in public and private sectors attribute to different approaches to patient counselling and education [26]. Given the impact of service operationalisation on practices, the overall goal of the study was to understand the factors influencing the obstetric providers’ role enactment in the service provision of the novel NIPT for DS. In line with the goal, the specific objectives of this study were to explore (i) the factors considered by participants when referring women for NIPT for DS in public and private healthcare sectors and (ii) their perceptions of the need to integrate NIPT into the current public antenatal service.

## Methods

The current study was part of a larger project to address ethical and public health implications in the clinical implementation of NIPT in Hong Kong. Using a sequential explanatory mixed-methods design [27], the first phase of a cross-sectional survey among DS screening providers assessed knowledge, attitudes and clinical experiences of NIPT compared to conventional screening and diagnosis. One of the significant findings was that obstetric providers in public sectors reported significantly more ethical concerns in the clinical implementation of NIPT than private providers, including women’s informed decision making, lack of consultation, unequal access to NIPT, and unnecessary use of resource (e.g., multiple DS screening tests) [18]. The finding addressed the importance of addressing system-level interventions in facilitating informed choice for reproductive autonomy, effective coordination between public and private sectors, and fairer resource allocation for better DS screening techniques, including an option of introducing NIPT in public antenatal care settings.

We subsequently developed a semi-structured in-depth interview guideline to qualitatively explore the findings from the quantitative phase and reasons behind the discrepant attitudes focusing on the role enactment among obstetric providers. Therefore, taking a qualitative approach, such as a phenomenological, narrative or grounded theory approach was deemed unnecessary. Instead, we used aspects of grounded theory, for example, purposive sampling, and saturation.

### Sampling and recruitment

A sample of potential participants for the interview was constructed from the survey respondents. They included obstetric providers who drew blood for NIPT analysis or referral for the service and those left their contact information for a follow-up interview. Among 610 obstetric providers invited, 327 returned the survey (response rate: 53.7%), and 123 (34.5%) consented to be contacted for the interview. We checked whether there were differences in demographics between those who agreed to be contacted or not, and found no significant pattern suggesting little concern for selection bias for the qualitative study. A quota-sampling matrix was constructed to invite participants for interview. This was based on profession (obstetrician and midwife), work sector (public and private sector), and whether or not potential participants provided NIPT. Simultaneous data collection and analysis enabled identification of emerging themes from the outset. Using a purposive sampling framework, we invited obstetric providers based on the study objectives and demographics. The sampling method is designed to maximise the variability of the sample in order to reach saturation (i.e obtain a comprehensive understanding until no new information is acquired), directing towards the generation and development of conceptual theory as opposed to creating a descriptive account [28]. In this study, we reached data saturation of the key themes around the seventeenth interview but decided to complete twenty interviews to ensure inclusion of participants according to our sampling matrix [29–31]. Written informed consent was obtained from all study participants. In-depth interviews were conducted in their offices lasting from 45 to 90 min, and all were completed between July and August 2014. Ethical approval was obtained from the joint university research ethics committee and clinical research ethics committee in Hospital Authority of Hong Kong.

### Semi-structured interview

The interview guide was first developed based on a review of the literature on NIPT and supplemented by the quantitative findings. Local experts on NIPT reviewed the questions to check whether the questions reflected the current antenatal care systems in Hong Kong and whether there were any leading questions. A pilot interview was conducted among obstetricians and midwives to ensure the language appropriateness. The final guide consisted of questions and probes under the following domains: (1) clinical experience of DS screening and NIPT, for example how obstetric providers enact their role in the consultation with pregnant women, (2) issues arising from decision-making for NIPT, such as barriers and challenges in consultation and referral, and (3) potential integration of NIPT in public sectors. Table 1 shows sample interview questions.

**Table 1:** Sample interview questions

1. Clinical experience of Down syndrome screening and NIPT	
• How do you introduce NIPT to women?	
• When you provide consultation on NIPT for women, how did women respond?	
• How do you find your clinical experience using the NIPT, compared to the current first-trimester screening and invasive prenatal diagnosis?	
2. Issues arising from decision-making For NIPT	
• What aspect of NIPT would women consider the most important?	
• Have you encountered any difficulties in the consultation? If so, what’re the barriers?	
3. Potential integration of NIPT in public sectors	
• Since the introduction of NIPT in your clinic, what changes have you observed in prenatal care?	
• What is your opinion about integrating NIPT in the universal screening pathway?	

All the interviews were conducted by the first author, an experienced qualitative researcher who was independent of the services in which the research was conducted, hence not known to the study participants. Having a single interviewer ensured consistency of the interview process. Following each interview, the researcher wrote memos reflecting on the interview process and any interview questions that needed refining or adding.

### Data analysis

Interviews were digitally recorded, translated into English and transcribed verbatim. NVivo 10 software was used to facilitate data storage, coding organisation, and allocating categories systematically. Data analysis was conducted using thematic narrative analysis [31] to identify.

(i) the factors considered by participants when referring women for NIPT for Down syndrome in public and private healthcare sectors and (ii) perception of the need to integrate NIPT into the current public antenatal service. Inductive analysis was conducted simultaneously to identify emergent themes apart from the concepts of role enactment. Transcripts were read repeatedly and broken down into meaningful units of texts by the first author. Codes were generated inductively by analysing content domains that emerged from the narratives, and codes were then clustered to form broader categories for presentation in the paper. These categories were further refined through member-checking by the research team members who have extensive expertise in qualitative health services research in this field. Additionally, to enhance the credibility of our interpretation of the findings, the first and second author regularly discussed the interviewer’s reflections on the interview process, and any personal assumptions and preconceptions, which may influence participants’ responses about genetic testing, termination of pregnancy, disability, parenting, and family.

## Results

Our study sample was diverse: 5 were male; 8 were obstetricians, and 12 were midwives; 13 worked in public sectors, and 13 provided referral and 7 performed blood draw for NIPT. The average years of obstetric practices were 13.3 years (SD = 9.1, range: 2–30 years) and provided NIPT service was 2.2 years (SD = 0.9 years, range: 1–4 years). Further details of the demographics of participants are shown in Table 2.

**Table 2:** Characteristics of interview participants

No	Gender/ Age	Education	Profession	Work Sector	NIPT Service	Years of Experience	
						Obstetrics	NIPT
1	m/ ≥ 51	Tertiary	Obstetrician	Private	Blood Draw	30	3
2	f/ ≤ 30	High School	Clinic Nurse	Private	Blood Draw	3	2
3	f/ 31–40	Tertiary	Midwife	Public	Referral	9	2
4	f/ 31–40	Tertiary	Obstetrician	Public	Referral	8	2
5	f/ 41–50	> Master	Midwife	Public	Referral	20	3
6	m/ 41–50	> Master	Obstetrician	Public	Referral	24	3
7	m/ 41–50	Tertiary	Obstetrician	Private	Blood Draw	20	4
8	f/ 31–40	Tertiary	Obstetrician	Public	Referral	7	2
9	f/ ≥ 51	Tertiary	Midwife	Public	Referral	20	1
10	m/ 31–40	Tertiary	Obstetrician	Public	Referral	10	2
11	f/ ≥ 51	Tertiary	Clinic Nurse	Public	Referral	2	1
12	f/ ≤ 30	≥ Master	Midwife	Public	Referral	3	2
13	f/ 41–50	Tertiary	Midwife	Public	Referral	20	1
14	f/ 41–50	≥ Master	Midwife	Public	Referral	25	3
15	f/ ≤ 30	High School	Clinic Nurse	Private	Blood Draw	9	1
16	f/ 41–50	≥ Master	Midwife	Private	Blood Draw	20	2
17	m/ 41–50	Tertiary	Obstetrician	Public	Referral	23	3
18	f/ ≤ 30	Associate	Clinic Nurse	Private	Blood Draw	3	3
19	f/ ≤ 30	High School	Clinic Nurse	Private	Blood Draw	5	3
20	f/ ≤ 30	Tertiary	Obstetrician	Public	Referral	4	1

*f* = female, m = male

We have identified four major thematic categories following analysis: (1) perceptions of information to facilitate women’s decisions about NIPT; (2) perceptions of clinical guidance needed on referral for NIPT; (3) private providers’ assumptions about the provision of public pre-test information for NIPT; and (4) perceptions of equitable access to NIPT.

### Perceptions of information to facilitate women’s decisions about NIPT

In the consultation on prenatal screening, the obstetric providers said that they mostly focused on clinical and procedural aspects of testing. They did not discuss the options available after screening such as termination of pregnancy (TOP) and health conditions of the affected unborn baby, which they consider as topics of pre-counselling for IPD. This principle was applied similarly to NIPT. Participants confirmed that the information they provided about NIPT was mainly factual, including NIPT being a blood test, its sensitivity, no risk of miscarriage, test limitations and the conditions screened. None of the participants believed the topic of TOP was essential pre-test information for NIPT, because they considered ‘NIPT is no more than screening’ and TOP as too sensitive to discuss at this stage.

We would not mention the term, *termination* of pregnancy explicitly but frame it in this way, like ‘would you like to continue the pregnancy or to have other thoughts?’ (midwife, public).

Participants said that women found to be at high-risk following screening often asked them for advice about NIPT, ‘should I take it or not?’ Participants explained that they were reluctant to give advice or engage in discussion about NIPT, particularly those from public sectors, because the decision to opt for NIPT should be the women’s. Participants often used terms such as ‘up to them’ and ‘their free choice’ implying their understanding of being able to influence women, hence hesitance to get involved in the decision-making process by avoiding discussion of NIPT:

My duty is to inform her with factual information about advantages and disadvantages of each test. We do not want to involve making a decision for private service with patients but give them liberty. (midwife, public).

Reluctance to discuss information about NIPT also appeared to be due to perceptions of their own lack of knowledge about cell-free fetal DNA sequencing technology. Obstetricians mostly obtained the information through scientific reports while midwives and clinic nurses primarily relied on informal training provided by workplaces, such as verbal informants and leaflets. Participants explained feeling unprepared to answer ‘hard questions’ from women:

Our level of understanding of NIPT is limited and very simple. I am afraid that women would not be able to receive proper information until they walk into the room and see a doctor. (clinic nurse, private).

Many participants in public sectors were concerned about providing information that may influence women’s decision. They understood the importance of providing more factual information to support women’s decision-making but were concerned that more in-depth conversations about NIPT could influence them. Also, while informing women about NIPT was considered important, participants acknowledged time constraints in public sectors and that NIPT was only available in private sectors, highlighting the issues arising from the overlap of public and private service provision:

NIPT is not part of the universal prenatal screening program. Unlike the private sectors that offer a test and incur a charge of consultation on women, time does not allow us [public providers] to give detailed consultation on prenatal screening for everyone. (obstetrician, public).

Participants also explained that in conversation with pregnant women, they explore personal values and understandings of NIPT, but time constraints hindered in-depth discussions on NIPT in public services. This lack of information relates to participants concerns about the lack of regulations in the use of NIPT in the private sector:

If women have access to commercial laboratory-based genetic test without a referral, I doubt whether laboratories have designated accredited persons to ensure women’s autonomy by full comprehension on testing procedure and outcomes, and their implications in their life. (obstetrician, public).

A provider in private sectors commented:

The laboratory is an analysis site that does not include consultation provided by doctors. Test analysis site requires a person to oversee, read, and interpret a report. I would be concerned if women left uninformed. (clinic nurse, private).

Participants envisioned that advance in DNA sequencing would lead to the availability of testing for an increasing number of genetic conditions, but worried about the insufficient availability of genetic counselling to enable women to understand the implications of test results and support subsequent decision-making:

Genetic technology has advanced quickly from basic research to clinical trials and commercialisation. It has dangerously left doctors unprepared for handling the broader scope of health conditions that can be detected by NIPT. In the local context we have insufficient counselling experts, we doctors are responsible for counselling, despite our unpreparedness. (obstetrician, public).

### Perceptions of guidance needed on the referral for NIPT

The current local health authority guidelines on DS screening do not specify the use of NIPT on the prenatal screening pathway. Participants believed that this further introduced challenges in the provision of antenatal care and confusion about the roles and responsibilities of healthcare professionals in public and private sectors:

The local authority has not yet addressed the use of NIPT under the existing system. We, public providers, were also *lost*. We did not know what to do with a referral. Many clinicians are offering NIPT. They may approach the test differently. I do not know how they do. I am concerned that patients fail to receive the testing service in a standard way. (obstetrician, public).

Furthermore, public providers gave specific details on the challenges, for example, who would be offered NIPT. The cut-off ratio for high-risk varies by laboratories and clinics. Clinicians had a different interpretation of risk.

The high-risk cut-off from routine screening ranges from 1:250 to 1:270. It highly depends on how a doctor interprets the result as understanding the idea of ‘risk’ differs. For example, would you refer a woman with a result of 1:251 to take an additional test, although she does not fall into ‘abnormal’ category, yet far from ‘safe’ zone? It varies across obstetricians. (obstetrician, public).

Participants said that many women asked a question about how NIPT was acknowledged in public services of prenatal testing and sought an opinion about which test among those available privately they should undergo. Public providers found it challenging to deliver neutral counselling and provide information in the absence of guidelines from the local public health authority.

Even after giving out all test information, women did not apprehend and wanted to seek a directive, actionable suggestion from me. They often asked me ‘what would you do in my position? I do not understand the options here.’ I felt challenged that I do not know how to respond and where should I refer to? (midwife, public).

Their advice was then that women should contact private clinics hoping that women would eventually receive comprehensive information about NIPT.

### Private providers’ assumptions about the provision of public pre-test information for NIPT

Unlike obstetric professionals in public services referring women for NIPT, obstetric professionals in private services providing NIPT showed more diverse opinions about NIPT. Two types of service approaches were found. Some private providers assumed that if a woman made a booking for NIPT with referral via public sectors, she must have had sufficient information to make an informed decision:

If a woman [from the public] calls and give explicit instructions for scheduling an appointment for NIPT, I would proceed to appointment booking directly. I would not provide the test information for those women unless they enquired what test they should take. (clinic nurse, private).

On the other hand, some private providers believed it was important to ensure women were making informed decisions about NIPT and, therefore, not to make any assumptions about information that may have already been provided or women’s understandings of this information:

Whether women come with or without a referral, we must reiterate the purpose of their visit and discuss testing options with them from the beginning. They receive clearer and better information from us than public sectors before meeting our doctor. (clinic nurse, private).

In addition to prior screening history and risk indicators, pre-test discussions by private providers covered the cost of NIPT. Participants explained that when there was no financial constraint for women, they preferred this test due to its higher test accuracy.

I would explain to women the price of each screening test and subsequent cost if the result is positive. The conventional screening is cheaper than NIPT. However, women could save more money if women take NIPT directly as primary screening in the first place. Taking NIPT as a contingent test upon receiving a positive test result would cost more money and time. (clinic nurse, private).

Private providers’ views towards informed consent were similar to their public counterparts – screening in no need for going in detail. Notably, some private providers said that taking NIPT as a secondary test, after negative result DS screening would not need a full informed consent procedure as they believed that the primary purpose of NIPT is psychological assurance.

Many women do not have questions, and just take a look at the consent form and give their signature. Probably, most laymen would think it is fine as long as the test provides them with an assurance and reduces their anxiety. (clinic nurse, private).

### Perceptions of equitable access to NIPT

The participants commented on scarce resources in the public antenatal care setting and acknowledged the vital role of private sectors in service delivery. Participants believed that the main reasons for women’s uptake of private services in addition to public services included the failure of timely access to public antenatal care and for reassurance. Accordingly, participants suggested that NIPT addressed needs:

Paying out of pocket for NIPT may be costly but very meaningful for women who missed the first-trimester tests and limited to second-trimester screening only. I learnt from my obstetric colleague, who was also a pregnant woman that the feeling after being reassured for abnormality-free results from routine screening and NIPT could be very substantial. (obstetrician, public).

Participants also highlighted that multiple visits to both public and private services for cross-monitoring (known as ‘doctor shopping’) was not uncommon in Hong Kong. They observed women who had taken NIPT, also opting for DS screening in public services, where they know the latter has lower accuracy. Such uptake of multiple screenings was believed to result in unnecessary screening and, hence, spending of scarce public healthcare resources. Some participants also raised ethical concerns about the high cost of NIPT, hence its availability only to those who could afford it. Participants believed the overlap between public and private services lead to an unjust healthcare service where less privileged women did not have equitable access to NIPT as a reassuring test and instead had to opt for tests with a risk of miscarriage:

NIPT is expensive. I believe that the public healthcare system cannot afford to support the cost of NIPT for every woman. How many ordinary families could afford this test without receiving a subsidy from the government? As a consequence, only rich people can take this test while others cannot afford the test and suffer more. (obstetrician, public).

A consensus among participants was prevalent that without proper regulations facilitating better integration of NIPT, the gap between public and private sectors was likely to widen.

## Discussion

The study identified the factors influencing their role enactment in the consultation and referral regarding the service provision of NIPT for DS in public and private healthcare sectors, including information for decision making, guide of referral for NIPT from public to private sectors, private sectors’ assumption and stance of NIPT pre-testing information before referral, and concerns about equitable access to NIPT. The lack of standard care was found to be related to not only the characteristics of a laboratory-private test but also the participants’ view that NIPT is similar to routine screening [32]. They used the same degree of informed consent procedure for DS screening in NIPT holding an opinion that a detailed discussion is unnecessary [10]. As the result of NIPT has no direct implication on whether the pregnancy would be continued or terminated, the obstetric providers in public sectors would not feel the need for the use of their scarce resource (e.g., time, information, and communication) to enhance the quality of NIPT service. This raises two important questions concerning the purpose of informed consent and informed decision-making for NIPT.

The goal of prenatal testing is to enhance reproductive autonomy, which is safeguarded by informed consent enabling women to fully understand the purpose, procedures, risk, and benefits of the test [33]. Discussion of TOP is only part of informed consent that covers women’s knowledge, norms, and values. However, the majority of providers in the study justified the depth of informed consent based on whether there should be a discussion on TOP or not; if no discussion on TOP, informed consent can be simple. This outcome-based informed consent disregards the real reasons for women to undergo the test – to know the condition of the unborn baby. Such an approach to informed consent is rather paternalistic as it ignores a core principle of whether or not a woman wants to discuss, not of whether or not TOP should be discussed. Some women prefer to discuss TOP after receiving the confirmation by IPD as early discussion would induce psychological distress, but some women prefer to discuss at an early stage. Withholding the discussion might undermine women’s capacity to recognise her choice of screening at the time of the decision-making.

While the literature on ethical issues of NIPT addresses the erosion of informed consent due to the eases related to the test [33], it fails to address the issue of referral between public and private sectors and how this relates to health professionals responsibility to ensure patients are making autonomous decision [34]. One of the salient themes in this qualitative study was providers’ reluctance to engage in decision-making for prenatal testing. Furthermore, nondirective counselling is recommended by obstetric professional societies [35]. A study with obstetric providers and patients in the United States found that the nondirective approach was delivered by an emphasis of ‘screening is optional’ and normalisation of screening ‘other women choose as well’ [36]. The study also noted that lack of in-depth discussions on prenatal screening was prevalent between providers and patients and therefore a lack of patient understanding was reported. Another qualitative study in Hong Kong also found that providers often delineated their roles and constructed their professional identity as a non-directive information provider [37].

There is an ethical dilemma that the ethos of ‘it is the patient’s choice’ often leaves pregnant women isolated and unsupported to make informed choices [38]. Supporting women to make informed choices about prenatal screening tests is especially important to address in countries with two-tiered healthcare systems, where the likelihood of women receiving information and professional support for decision-making from either is low. Public providers only offer tests subsidised by public funding but may feel morally responsible for informing pregnant women about the availability of public tests to provide a ‘better’ antenatal service. Further research is needed to devise a culturally appropriate informed decision-making guideline for NIPT specific to the healthcare system [39, 40]. Our finding shows that there is a need for guidelines for public services on making referrals to private services, to provide an optimal antenatal service for all women.

There are practical reasons for the need of guidance. Since NIPT was first commercially launched in late 2011, professional societies’ policy and recommendations have been changed according to scientific evidence. Several countries currently consider widening access of NIPT subsidised by the national funding. In the UK, the National Health Service (NHS) intends to incorporate it as a contingent screening test in a publicly funded program for all women screened high-risk from first-trimester screening starting from 2018 [41]. In the Netherland, the Health Council plans to adopt the test for all pregnant Dutch women [42]. Meanwhile, the advance of cell-free fetal DNA sequencing technology is expanding gene panels to detect a broader range of fetal conditions. Commercial companies offer NIPT for conditions, including sex chromosome aneuploidy and a few micro-deletions syndrome, despite the limited of robust evidence and assessment of risk-benefit [43]. In response to these developments, the national framework or regulatory guideline of NIPT is essential for the quality care and patient safety reflecting societal norms and healthcare system [44].

There were different patterns of responding to the issue of affordability of NIPT. In private sectors, if women were found to afford the test, NIPT was recommended as it would shorten the clinical pathway for DS diagnosis and might reduce pregnancy-related anxiety. In public sectors, the providers often faced a question of whether they should refer a woman to a better option of NIPT in private sectors and in doing so how they engage in discussions about the benefits over the cost. This relates to a concern about fair treatment. In ethics of distributive justice, unequal access to medical intervention is permissible if patients are not harmed significantly without it. For example, although the quality of public education might not be good as, perhaps poorer than, private education, we would not say public education system is unjust. The current DS screening in Hong Kong has a rigorous internal and external quality assurance programme that identifies 93% DS affected pregnancies suggesting using NIPT as primary screening may be redundant and surplus of antenatal care resources [45]. However, for the justice issue of women in public sectors not being harmed more than those in private, NIPT needs to be introduced in public sectors to reduce the fetal loss by IPD procedure. For ‘low-risk’ women, the consultation and informed consent for DS screening need to be enhanced as it plays a critical role in ensuring women’s autonomy and reduce maternal anxiety.

### Limitations and strengths

There are several limitations. The study did not assess the degree of participants’ knowledge and views of NIPT, which might be associated with perceived barriers to providing the test. The study did not explore institutional policy aspects of NIPT by interviewing the hospital managers. Further studies among other stakeholders are needed. Nevertheless, our study highlights important points that could inform policy development about the integration of NIPT within public services in HK and other countries.

## Conclusions

NIPT is a good example of why the public and private sectors should work together to improve quality care for women. As one of the most rapidly introduced and widely used out-of-pocket tests, many countries are under consideration of introducing it into the public healthcare system for pregnant women who are deemed at high risk for DS. The potential conflicts of roles and responsibilities in public and private sectors have been observed – how the healthcare system and organisational factors influence the enactment of obstetric providers’ role in the consultation of NIPT. In response to policy discussion of clinical adoption, it is necessary to minimise the discrepancy in knowledge and attitudes of NIPT between the sectors by providing continuing medical education on the advance of cell free fetal DNA sequencing technologies and standardised protocol of NIPT procedure, enacting a clear referral and counselling guideline, and establishing monitoring system for quality assurance across the sectors. Not all pregnant women are eligible to receive publicly subsidised NIPT and there are many laboratory companies offering the test at varying costs. Thus, the development of practice guideline is essential to enable better coordination NIPT between the two sectors services to enable women to make informed choices about the uptake of NIPT privately. Such guidelines should not be drawing the boundary between two sectors but how they should collaborate, especially how private sectors can supplement the limited resources in public sectors to fill the gaps and address the needs of pregnant women.

## Abbreviations

DS: Down syndrome
IPD: Invasive prenatal diagnosis
NHS: National Health Service in the UK
NIPT: Non-invasive prenatal testing
TOP: Termination of pregnancy
